# Evaluating Winter Vitamin D Supplementation and Deficiency in a Tier 4 CAMHS in Patient Unit: A Three-Cycle Audit

**DOI:** 10.1192/bjo.2025.10634

**Published:** 2025-06-20

**Authors:** Bryony Mills, Liam Young

**Affiliations:** Lavender Walk, General Adolescent Unit, London, United Kingdom

## Abstract

**Aims:** Vitamin D is an essential nutrient for adolescents, playing a crucial role in bone health, immune function, and neurodevelopment. Its synthesis is highly seasonal, with production significantly reduced during the winter months due to limited sunlight exposure. This effect is further exacerbated in CAMHS inpatients, who may spend extended periods indoors with even less access to natural light. Recognising this, the National Institute for Health and Care Excellence (NICE) recommends vitamin D supplementation for at-risk groups, including those with restricted sun exposure. Given the high prevalence of vitamin D deficiency among adolescents with mental health conditions, all CAMHS inpatients should be offered maintenance vitamin D supplementation from November to March. This audit aimed to assess compliance with this standard on a Tier 4 inpatient unit.

**Methods:** A three-cycle audit conducted monthly from November to February, assessing vitamin D prescription rates and serum levels in CAMHS patients. Data was collected through single-point reviews to evaluate prescribing trends and deficiency management. Interventions included 1:1 psychoeducation from a doctor and a poster in the clinic room promoting opportunistic serum vitamin D testing and supplementation.

**Results:** The audit results show a substantial improvement in vitamin D prescribing over three cycles. Initially, only 1 out of 10 inpatients received supplementation (18/11/2024). By the second cycle (06/01/2025), this increased to 9 out of 11 patients, with a notable rise in prophylactic prescriptions (7 patients). By the third cycle (05/02/2025), the prescribing rate remained high (9 out of 10 patients), with 7 receiving prophylaxis and 2 on treatment doses.

Regarding vitamin D level monitoring, the number of patients with levels checked within the past three months remained consistent at 5 across all three cycles. However, the number of patients without recent vitamin D levels fluctuated, increasing to 6 in January before returning to 5 in February. These findings highlight an improvement in prescribing practices but suggest a need for increased consistency in vitamin D level monitoring.

**Conclusion:** The audit showed that the 1:1 psychoeducation intervention provided the most benefit, with notable improvements from cycle 1 to cycle 2. However, increasing vitamin D testing is needed to optimise supplementation and prevent sub-therapeutic treatment in CAMHS patients, potentially shifting from opportunistic to a more proactive approach.

